# Hydrodynamic Cavitation on a Chip: A Tool to Detect Circulating Tumor Cells

**DOI:** 10.1021/acsami.2c12356

**Published:** 2022-09-01

**Authors:** Ilayda Namli, Seyedali Seyedmirzaei Sarraf, Araz Sheibani Aghdam, Gizem Celebi Torabfam, Ozlem Kutlu, Sibel Cetinel, Morteza Ghorbani, Ali Koşar

**Affiliations:** § Faculty of Engineering and Natural Sciences, Sabanci University, 34956 Tuzla, Istanbul, Turkey; ‡ Sabanci University Nanotechnology Research and Application Center, 34956 Tuzla, Istanbul, Turkey; # Center of Excellence for Functional Surfaces and Interfaces for Nano-Diagnostics (EFSUN), Sabanci University, Orhanli, 34956 Tuzla, Istanbul, Turkey

**Keywords:** liquid biopsy, circulating tumor cells, CTCs detection, hydrodynamic cavitation on chip, lab on a chip, heterogeneous nucleation

## Abstract

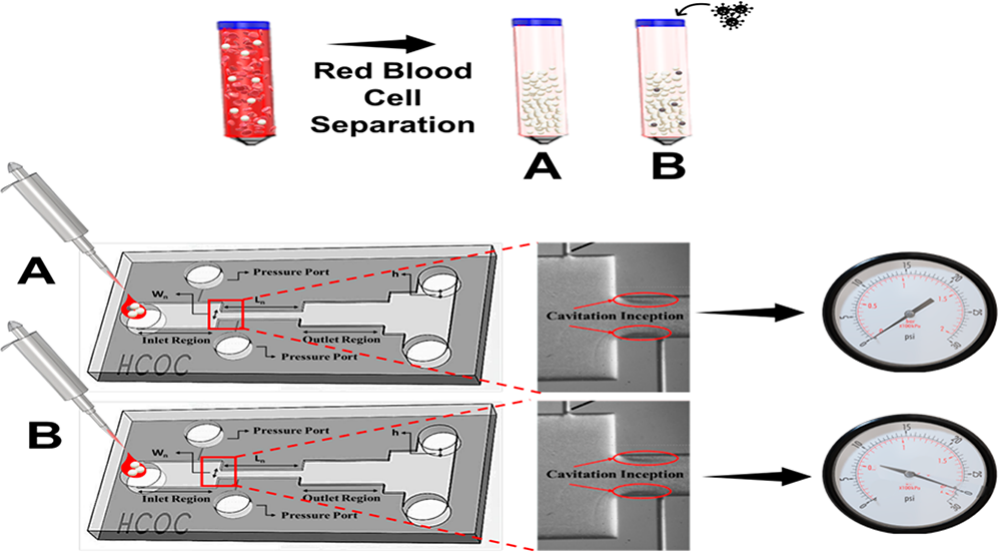

Circulating tumor cells (CTCs) are essential biomarkers for cancer diagnosis. Although various devices have been designed to detect, enumerate, and isolate CTCs from blood, some of these devices could have some drawbacks, such as the requirement of labeling, long process time, and high cost. Here, we present a microfluidic device based on the concept of “hydrodynamic cavitation-on-chip (HCOC)”, which can detect CTCs in the order of minutes. The working principle relies on the difference of the required inlet pressure for cavitation inception of working fluids when they pass through the microfluidic device. The interface among the solid/floating particles, liquid, and vapor phases plays an important role in the strength of the fluid to withstand the rupture and cavitation formation. To this end, four experimental groups, including the “cell culture medium”, “medium + Jurkat cells”, “medium + Jurkat cells + CTCs”, and “medium + CTCs”, were tested as a proof of concept with two sets of fabricated microfluidic chips with the same geometrical dimensions, in which one set contained structural sidewall roughness elements. Jurkat cells were used to mimic white blood cells, and MDA-MB-231 cells were spiked into the medium as CTCs. Accordingly, the group with CTCs led to detectable earlier cavitation inception. Additionally, the effect of the CTC concentration on cavitation inception and the effect of the presence of sidewall roughness elements on the earlier inception were evaluated. Furthermore, CTC detection tests were performed with cancer cell lines spiked in blood samples from healthy donors. The results showed that this approach, HCOC, could be a potential approach to detect the presence of CTCs based on cavitation phenomenon and offer a cheap, user-friendly, and rapid tool with no requirement for any biomarker or extensive films acting as a biosensor. This approach also possesses straightforward application procedures to be employed for detection of CTCs.

## Introduction

Despite contemporary advances in healthcare technologies, cancer globally remains one of the prevalent causes of mortality. It was projected that 9.1 million individuals would be diagnosed with breast and colorectal cancer in 2070, a 131% rise from 2018, according to modeling the future burden of cancer by Soerjomataram et al.^1^ Cancer morbidity and mortality could be avoided if cancer is detected at an early stage. This is highly important not only for prognosis but also for patient stratification and treatment strategy.

Circulating tumor cells (CTCs) have enormous potential as a biomarker for early cancer diagnosis, prediction, and prognosis.^2^ Detection of CTCs migrating from primary tumors into the bloodstream has received more attention during recent years because they assist in the early detection and cancer therapy of patients.^2^ Previous studies reported the existence of CTCs in the early stages of the cancer and related it as an indication of the disease progression.^3^ Several studies stated that early CTC detection is a promising sentinel of tumor development.^4−6^ Different CTC technologies were developed to detect, enumerate, or isolate these rare cells.^4^ The key features of a CTC analyzer device are repeatability, reliability, rapidity, cost efficiency, sensitiveness, and user friendliness, which must be addressed before commercialization. The main challenge is that CTCs are rare cells, whose incident number in the peripheral blood circulation compared to other hematologic cells is 10^0–3^/10^5–9^ in mL.^4,5,7^ To address this problem, CTC technologies employ two different strategies involving immunoaffinity and biophysical properties of CTCs. Immunoaffinity utilizes the presence of distinct protein biomarkers such as EpCAM (epithelial cell adhesion molecule) on the surface of target cancer cells to capture them onto a desired location.^4^ The CellSearch system is the only Food and Drug Administration (FDA) approved positive enrichment immunoaffinity technology so far for some cancer types, such as breast, prostate, and colorectal cancer, and separates the CTCs magnetically using the functionalized ferrofluid nanoparticles with EpCAM antibody. However, levels (presence) of EpCAM protein expression on the CTCs vary, and the CTCs expressing low-level EpCAM could be lost during the enrichment process.^8^

On the other hand, label-free techniques including filtration, centrifugation, streamline sorting, and dielectrophoresis, which are based on the biophysical properties of CTCs, including size, deformability, density, and polarizability, have been investigated in the literature.^9−11^ For example, Sollier et al. reported that the size of CTCs collected with a label-free microfluidic device ranged from 12 to 25 μm in diameter, while this amount for leukocyte was 2–14 μm.^9^ Although size-based collection or detection approaches could have disadvantages such as low purity, clogging, and limited volume, vortex technology provides screening in a short time, high purity, and application availability in different cancer types. Furthermore, ApoStream is a system based on the dielectrophoresis technology and utilizes the difference of electrical response of various cells depending on the composition and morphology of the cell type.^12^ This system is able to test a 10 mL sample within an hour while capturing viable cells for post-processing analysis.^4^ In the highlights of this study as well as of several other studies, it could be stated that size-based techniques offer a fast and simple approach. There are also new methods for CTCs’ detection or isolation, which are under investigation and development, such as direct imaging modalities.^13^ While hydrodynamic cavitation (HC) has been proven as a versatile phenomenon in biomedical applications, the detection of CTCs based on HC inception has not been investigated so far in microfluidic devices.

Cavitation is a process of nucleation, growth, and collapse of bubbles filled with vapor or gas. When the static pressure drops below the saturation vapor pressure of the liquid, cavitation bubbles appear in the liquid. In HC, the rapid pressure drop can be accomplished within a microfluidic device containing a microflow restrictive element called a microchannel. Cavitation inception occurs when the first bubbles appear inside the flow restrictive element, and the local pressure, at which cavitation incepts, is called the “cavitation inception pressure”. Although it is homogeneous nucleation and thermal motion which cause the generation of the bubbles from the microscopic voids within the medium, the impurities and weakness points at the boundaries or within the liquid, acting as the features of heterogeneous nucleation, substantially affect the inception pressure (by reducing it).^14^ This change in the inception pressure reduces the required energy upstream of the microchannel entrance within the microfluidic device. HC has applications in biomedical engineering for treatment and diagnosis purposes, and its application areas have been continuously widening. As a pioneering study, Itah et al. showed HC killing prostate cancer cells and that HC could be used to ablate tumor tissues.^15^ Another study reported that prostate and bladder cancer tissues were significantly damaged even at lower pressures within less than 15 min once the bubbles collapsed and self-destructed with a flexible cystoscope designed and developed for HC-based therapy.^16^ Although HC has a destructive effect on living tissues, Gevari et al. referred to the diagnostic approach of HC, which shows that bubbles generated different effects and different modes of damage on a variety of immobilized cancer cell lines, indicating its potential for identifying cancer cells.^17^ In another study, the irregular shape of *Salmonella typhimurium* bacteria in the working fluid served as a solid interface, which favored earlier cavitation generation (lower cavitation inception pressure).^18^ The amount of decrease in the inception pressure could be related to the size of the particles, which motivated the authors to distinguish CTCs from other homologous cells in this study.

The designed microfluidic device in this study is based on CTC detection from the biological fluids and blood samples with the “hydrodynamic cavitation-on-chip (HCOC)” approach. Accordingly, HC is generated in a previously fabricated microfluidic device^19^ by triggering a rapid pressure drop using a micro-orifice.^20−22^ The concept of the detection system is based on a comparison of the cavitation inception pressures of three different working fluids. Cell culture medium, Jurkat, CTC, and Jurkat + CTC groups were employed as working fluids through the device. In addition, the effect of structural sidewall roughness elements on cavitation inception was evaluated. The system acquires pressure values, while three different working fluids separately pass through the HCOC microfluidic device, at cavitation inception, which is captured by utilizing the shadowgraph technique via a high-speed camera. Moreover, blood samples collected from healthy volunteers and CTC-spiked blood samples were tested in the HC setup. The experiments revealed that the Jurkat + CTC group and CTC-spiked blood sample had a lower cavitation inception pressure than the groups Jurkat and blood samples without CTC, respectively. Therefore, CTC enriched content led to considerably earlier cavitation inception. Our results prove that the proposed cavitation inception-based tool could be a feasible and promising technique to rapidly detect the presence of CTCs.

## Experimental Section

### Cell Culture Sample Preparation

**Figure 1:**
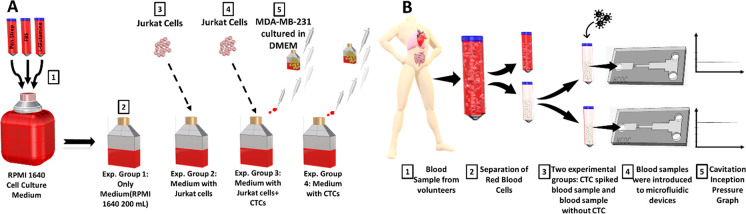
(A) Schematic of cell culture preparation before the experiments: (1) The group including only RPMI 1640 cell culture medium, (3–4) adding Jurkat cells on a flask, (5) seeding MDA-MB-231 cells, cultured in DMEM previously, on a flask. (B) Schematic of the blood sample preparation before cavitation tests. (1) Blood samples were collected from volunteers into sample tubes. (2) Red blood cells were lysed, centrifuged, and discarded. (3) Remaining samples were divided into two groups considering cell concentration, and CTCs were spiked into one of the groups. (4) Each sample was introduced to different microfluidic devices with exactly the same configuration. (5) Cavitation inception pressures were recorded for each group, and graphs were plotted.

Studies have shown that Jurkat cells correspond to the cell line most similar to leukocytes in terms of size and elastic properties. Therefore, Jurkat cells are widely used to mimic white blood cells in the studies involving CTCs.^23,24^ In this study, human breast adenocarcinoma cell line MDA-MB-231 [American Type Culture Collection (ATCC) number HTB-26] and Jurkat human acute T lymphocyte leukemia cell line (ATCC-Clone E6-1 number TIB-152) were employed. Roswell Park Memorial Institute Medium 1640 (RPMI 1640, P04-17500), Dulbecco’s modified eagle medium (DMEM, P04-03500), and fetal bovine serum (FBS, P30-3306) for cell culture were purchased from PAN-Biotech (Aidenbach, Germany). Penicillin-streptomycin solution, l-glutamine, and trypsin EDTA solution C (0.5%) were obtained from Biological Industries (Beit HaEmek, Israel). MDA-MB-231 cells were cultured in DMEM, whereas Jurkat cells were cultured in RPMI 1640. Both basal cell culture media were supplemented with 10% heat-inactivated fetal bovine serum (FBS), 100 units/mL penicillin, 100 μg/mL streptomycin, and 100 μg/mL l-glutamine. 100× nonessential amino acids MEM-NEAA solution (Gibco, 11140035) was also added into DMEM. Cells were incubated at 37 °C in a 5% CO_2_ humidified incubator. When the confluency of MDA-MB-231 cells reached 90%, cells were passaged after detaching from the surface with 0.25% trypsin/EDTA solution. Since Jurkat cells are nonadherent cells, they were passaged with fresh medium in appropriate proportions without trypsin treatment. The number of dead cells was measured by using trypan blue before each experiment, and the live cell percentage was always kept above 95%. Unless the live cell concentration was lower than 95%, the removal of deal cells with centrifugation was not applied. Four experimental sample groups were prepared (Figure 1): the first group contains only medium (RPMI 1640), while the second group contains Jurkat cells within the medium (Jurkat group), the third has Jurkat cells and cancer cell line (Jurkat + CTC group), and the last one contains Jurkat cells + cancer cell line (CTC group). There is 200 mL of RPMI 1640 medium in the no cell containing group. For the Jurkat cell group, the concentration of Jurkat cells was adjusted to 1 × 10^6^ cells/mL in a total of 200 mL. The CTC concentration was 300 cells/mL in a total of 200 mL medium. Similarly, the Jurkat cells + CTCs group was prepared in 200 mL of total cell culture volume with the Jurkat cell concentration 1 × 10^6^ cells/mL and CTC concentration 300 CTCs/mL. The same procedure was followed for different concentration tests using 1 × 10^6^ cells/mL Jurkat cells and 100 CTCs/mL and 50 CTCs/mL, respectively. The confluency of CTCs cultured in DMEM was as high as (1–2) × 10^6^/ mL. Therefore, a very small volume of DMEM containing CTCs needed to be added into RPMI 1640 to obtain 300 CTCs per mL in RPMI. At the end, the percentage of the DMEM volume in RPMI was approximately 0.001%, which was less than the error of the detection system. Hence, the amount of DMEM including cancer cells did not impact the measured cavitation inception of the group with CTC added in RPMI 1640.

### Blood Sample Preparation

The study protocol was approved by Yeditepe University KAEK (2012-KAEK-70). Blood samples were collected from healthy volunteers into vacutainer tubes containing the anticoagulant EDTA. After completing cell culture tests, processed blood samples were divided into three groups considering cell number per mL. Blood samples of the first (N1) and second (N2) groups do not include CTC and were collected from different volunteers, which resulted in different blood characteristics. While blood samples of the second and third groups were collected from the same volunteers, only the third group contains CTCs. Peripheral blood samples (10 mL in K-EDTA) were diluted 1:3 with 1× red blood cell lysis buffer (87.4 g/L NH_4_Cl, 10 g/L KHCO_3_, 1 mM EDTA for 10× lysis buffer stock). The mixture was incubated at room temperature for 10 min with gentle shaking. Then it was centrifuged at 2000 rpm for 10 min and repeated twice with the above-mentioned buffer to remove all red blood cells. White blood cells, including PBMCs, monocytes, and T and B lymphocytes, were obtained and resuspended with RPMI. Cell concentrations per mL were counted using a Thermo Fisher Countess II Automated Cell Counter.

### Microfluidic Device Design, Fabrication, and Experimental Procedure

#### Fabrication Method and Device Configuration

The microfluidic device consists of a patterned silicon wafer substrate bonded by a glass lid for the sake of visualization. The process flow to fabricate the device is summarized in this section and represented in Supporting Information 2. A double sided polished silicon wafer with a 500 nm silicon oxide layer was used. For the chips with sidewall roughness elements, an electron beam lithography step was performed to pattern the spin coated 500 nm ZEP520A photoresist. Then a deep reactive ion etching (D-RIE) step was performed to etch 500 nm silicon oxide, and the photoresist was removed. For opening the channels and ports, the thickness of the AZ-ECI photoresist used in photolithography was 2 μm, and the energy required for this thickness in photolithography was 320 mJ/cm^2^. After this step, the photoresist remaining on the surface was removed with a resist stripping step. In the second lithography process, a 2 μm thick photoresist was applied, and dry etching was followed to etch the SiO_2_ layer by a second photomask for opening the inlet, outlet, and channel. Afterward, the D-RIE procedure was applied to etch the substrate along a distance up to 330 μm. The coated resist was removed entirely in this step. Then, the etching process continued with the bottom side of the substrate. However, before the second D-RIE, the silicon substrate was coated with 2 μm Ti and Al layers on the bottom side so that the sample wafer could withstand applied stress in etching. Subsequently, residual SiO_2_ and Al, Ti layers were eliminated by a wet etching process. In the final step, the silicon substrate was bonded to Borofloat-33 glass by anodic bonding.

**Figure 2:**
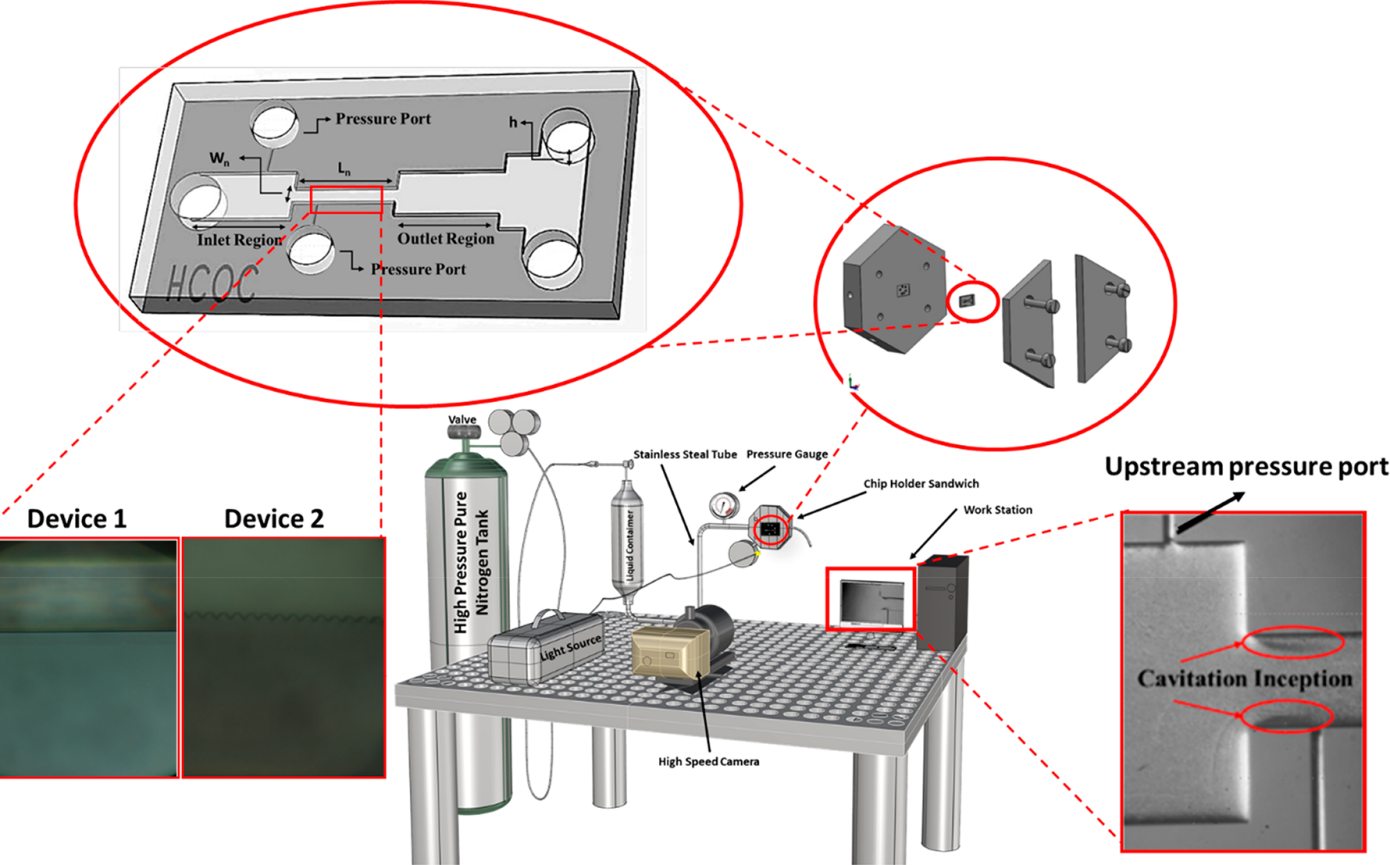
Experimental setup and microfluidic chip details. The cavitation inception is detected using the high-speed camera, and the pertinent pressure to the instance of cavitation inception is recorded as the cavitation inception pressure.

The microfluidic device (HCOC) used in this study mainly consists of an inlet, microchannel, and extension part designed on a silicon wafer (Figure 2). A pressure port is extended into the inlet region just upstream the microchannel’s entrance. The flow entering the device through the inlet encounters the microchannel, passes through the channel, and continues through the extension part. When the working fluid reaches the microchannel, which has a narrower cross-sectional area compared to the inlet region, a sudden pressure drop occurs at the vena-contracta region of the microchannel inducing cavitation inception. Finally, the fluid reaches the exit and leaves the microfluidic device. In this study, two microfluidic devices with the same configuration and different roughness properties were used. The first device has no surface or sidewall roughness (Chip-wo-R), while the second device has sidewall roughness (Chip-R). The total length of both microfluidic devices is 6000 μm. The width of the inlet region as well as the outlet region of the devices is 900 μm, and the microchannels’ width is 300 μm (Tables 1 and 2).

**Table 1:** Geometrical Properties of the Microfluidic Device without Roughness (Chip-wo-R)

Physical Configuration (Chip-wo-R)	Range
Microchannel length (L_n_)	2000 μm
Microchannel width (W_n_)	300 μm
Microchannel depth (h)	70 μm
Inlet/Outlet region length	2000 μm
Inlet/Outlet region width	900 μm
Length of the roughness elements (L_R_)	0
Height of the roughness elements (H_R_)	0

**Table 2:** Geometrical Properties of the Microfluidic Device with Roughness (Chip-R)

Physical Configuration (Chip-R)	Range
Microchannel length (L_n_)	2000 μm
Microchannel width (W_n_)	300 μm
Microchannel depth (h)	70 μm
Inlet/Outlet region length	2000 μm
Inlet/Outlet region width	900 μm
Length of the roughness elements (LR)	1/3L_n_
Height of the roughness elements (HR)	0.01W_n_

#### Experimental Setup of the Microfluidic System (HCOC)

The experimental setup for the HC test rig includes a high-pressure nitrogen tank (Linde Gas, Gebze, Kocaeli), liquid container (Swagelok, Erbusco BS, Italy), stainless steel tubes (Swagelok, Erbusco BS, Italy), and chip sandwich (holder). A CMOS high-speed camera (Phantom VEO-710L) with a resolution of 1280 × 800 pixels and pixel size of 0.02 mm was employed to visualize cavitation inception inside the microfluidic device during tests. A macro camera lens (type K2 DistaMax) with a focal length of 50 mm and an f-number of 1.2 was used. The high-pressure pure nitrogen tank was connected to the container, allowing the liquid inside the container to move through the tubing toward the device. The microfluidic device was connected to the pressurized tubing through a sandwich holder, which has an aluminum micromachined substrate and two transparent Plexiglass lids pushing the chip to the substrate by the means of screws shown in Figure 2. The sandwich holder has an inlet port, an outlet port, and a pressure gauge port (Omega, the USA with an accuracy value of ±0.25%) (shown in Figure 2).

### HC-On-Chip (HCOC) Experiments and CTC Detection Protocols

#### Cavitation Inception Measurement Procedure of Different Working Fluids

Hydrodynamic cavitation occurs when an upstream pressure large enough to initiate phase change is exerted to the fluid entering the flow restrictive element of the HCOC. The inception cavitation is identified using the shadowgraph technique, which differentiates the emergence of cavitation by high-speed recording of the reflection of the light source over the silicon substrate. At inception cavitation, a dark shadow of the gaseous region at the vena-contracta of the microchannel appears as could be seen in Figure 2. Thus, cavitation inception pressure values were assessed based on the measurement of the upstream pressure in the inlet region pressure port of the system at cavitation inception. Two pressure gauges at different locations were used to verify the measurements in order to avoid the errors caused by clogging at the inlet region pressure port. One pressure gauge was located just upstream of the inlet port of the sandwich, whereas the other pressure gauge was connected to the pressure port within the inlet region of the microfluidic chip through the sandwich holder. After each test, the chips were cleaned by the protocol stated in Supporting Information 1 and were reused for the next trial. A DI water test was performed as the reference value for the cleaning procedure before testing the other samples. The samples were introduced to the system by gently infusing them into the liquid container using pipettes. Four experimental groups, namely RPMI 1640, Jurkat, Jurkat + CTC, and CTC, were tested. A previously prepared 200 mL RPMI 1640 medium with no cells was tested, and the value for cavitation inception was recorded. Afterward, the group containing a certain amount of Jurkat cells in 200 mL RPMI 1640 medium was introduced to the system, and the inception cavitation was observed. Finally, the inception pressure of the Jurkat cells with the concentration of 300 CTCs/mL in 200 mL RPMI 1640 medium and an inception pressure of 300 CTCs/mL in 200 mL RPMI 1640 was recorded. The tests with all the groups were repeated for eight times as biological replicates using a unique unused sample for each trial. After complete sterilization of the experimental setup, blood samples were introduced, respectively. After each experiment, the microfluidic device was changed with another device having the same configuration with the previous one. Between subsequent experiments, the setup was cleaned and sterilized as stated in Supporting Information 1.

#### Cavitation Inception of Medium with Different Number of CTCs

Besides the billions of red blood cells and millions of white blood cells in the blood, CTCs are 1–10 per mL of blood.^25^ Therefore, CTCs, which are very rare in the blood, are very difficult to detect. Also, to evaluate the capability of the device in enumerating the number of CTCs, in addition to the cavitation inception pressure of Jurkat + 300 CTCs/mL, the inception pressures of Jurkat + 100 CTCs/mL and Jurkat + 50 CTCs/mL were also recorded for at least three times. The detection of enriched CTCs (different number of CTCs in mL) was based on hydrodynamic cavitation.

### Characterization Methods.

#### Scanning Electron Microscopy (SEM)

The samples were subjected to an SEM sample preparation protocol (see Supporting Information), and scanning electron microscopy offered further evaluation on cell morphology, including size and shape. The prepared samples were coated with three layers of Au/Pd, and the cells were observed using field emission scanning electron microscopy (FESEM, LEO Supra VP-55). The accelerating voltage was kept under 3 kV, and the working distance was in the range of 8 to 10 mm.

#### Cell Staining

DILC18(3) fluorescent dye (Life Technologies, D3911) was used to stain MDA-MB-231 and Jurkat cells. 2.5 mg of dye was added per mL of dimethyl sulfoxide (DMSO) (Santa Cruz, cs-358,801) to prepare a stock solution. The cells were resuspended in PBS (Gibco, 20,012-019) with 3% FBS at a concentration of 1,000,000 cells/mL. Stock dye was introduced at a 5 μL/mL cell suspension ratio. The stained MDA-MB-231 cell line was incubated for 20 min, while the Jurkat cell line was incubated for 5 min at 37 °C. Afterward, the cells were rinsed with PBS until the supernatant became totally clear.

#### Statistical Analysis

All experiments were conducted at least in triplicate. The mean values, standard deviations, and standard error values of all obtained results were reported. Relationships among groups were analyzed using one-way ANOVA (SPSS 12.0, SPSS GmbH, Germany) [Newman–Keuls multiple comparison test (* p\0.05, ** p\0.01, *** p\0.001)].

### Results

#### Cavitation Inception

**Figure 3:**
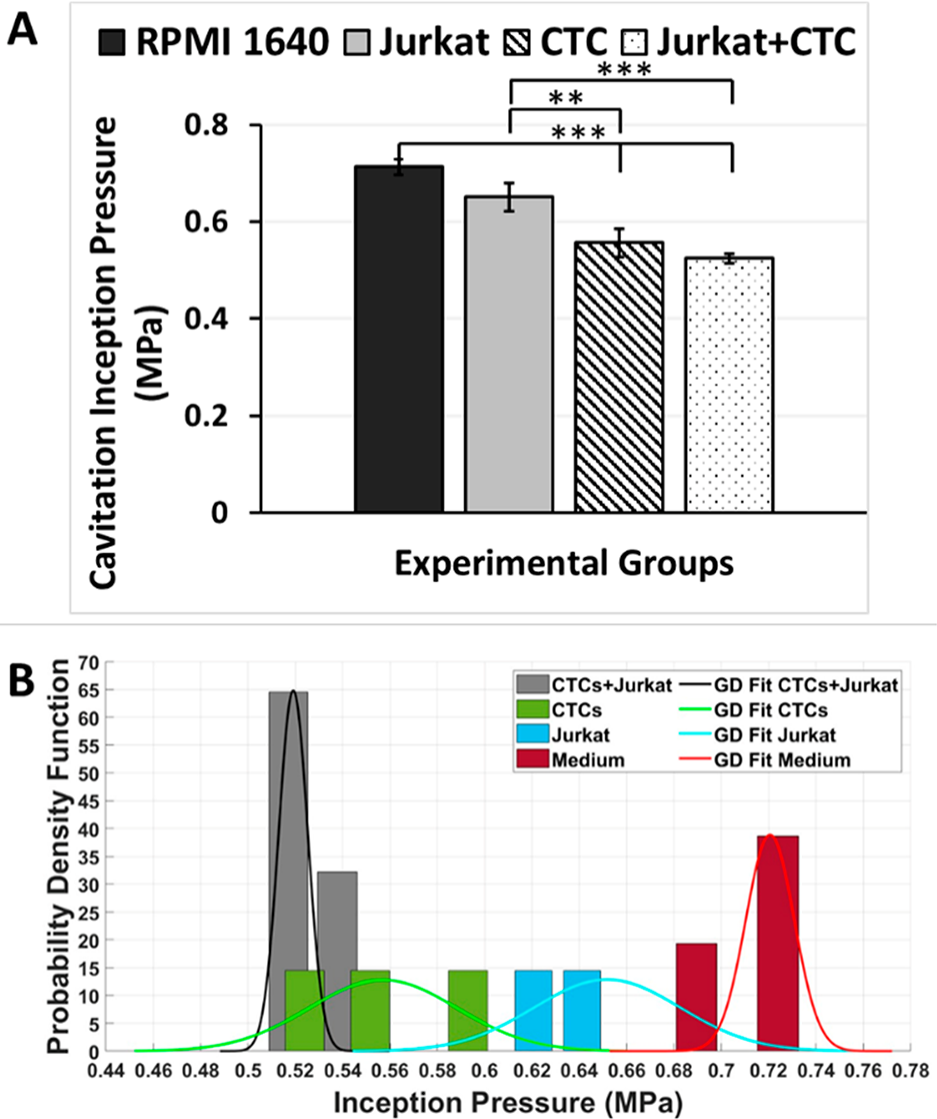
(A) Cavitation inception pressures of different working fluids in the microfluidic device without roughness (Chip-wo-R). The working fluids are RPMI 1640 cell culture medium, Jurkat, CTC, and Jurkat + CTC. Error bars indicate standard deviation. B) Probability distribution profile of medium, medium with Jurkat cells, and medium with Jurkat cells + CTCs in terms of cavitation inception pressure. The inception pressure data were obtained eight times for each sample, and a nonlinear fit was performed in MATLAB to show the inception pressure for each sample as a Gaussian distribution.

According to the results obtained from the tests for the first microfluidic device (Chip-wo-R) without roughness, RPMI 1640 (control group) was observed to have a high inception pressure compared to the Jurkat and Jurkat + CTC groups (Figure 3). In this case, the Jurkat cell concentration is 1 × 10^6^ cells/mL for the Jurkat and Jurkat + CTC groups, while the CTC concentration is 300 CTCs/mL for the CTC group and Jurkat + CTC. Hence, the ratio of CTC to Jurkat is 0.0003. RPMI 1640 leads to inception at 0.71 MPa ± 0.02, while the Jurkat group inception pressure is 0.65 MPa ± 0.03, and Jurkat + CTC leads to inception at 0.52 MPa ± 0.01. When the inception pressures of the fluid with Jurkat and fluid with Jurkat + CTC are compared, the inception pressure of the group with CTC exhibits a statistically significant decrease. Once the cavitation inception pressures of the different experimental groups are analyzed (Figure 3), it is evident that there is a significant difference between the group containing only RPMI 1640 and the Jurkat + CTC group. These results prove that when CTCs are added to the cell medium, the cavitation inception pressure is significantly reduced. Thus, the group containing cancer cells can be easily identified. Moreover, the inception pressure of the CTC group is 0.56 MPa ± 0.03.

Figure 3B shows the probability distribution of pressure values corresponding to different groups. Accordingly, the pressure range of the Jurkat + CTC group is between 0.5 and 0.54 MPa. The peak pressure value of the Jurkat + CTC group is approximately 0.52 MPa. This pressure value does not coincide with the Jurkat pressure distribution. On the other hand, the interval of Jurkat cells is broader than in the Jurkat + CTC group. The peak pressure of the Jurkat group is approximately 0.65 MPa. There is not an overlap area between the intervals of the Jurkat group and the Jurkat + CTC group. Besides, the cell medium interval range is between 0.65 and 0.77 MPa. It is seen that the area where the group Jurkat overlaps with the medium group is significant, indicating that Jurkat cells do not make any striking difference in inception values. However, there is no interval coincidence between the Jurkat + CTC group and group medium while there is a tiny overlap area between CTC and group medium, which demonstrates that groups with CTCs have a noticeable difference.

**Figure 4:**
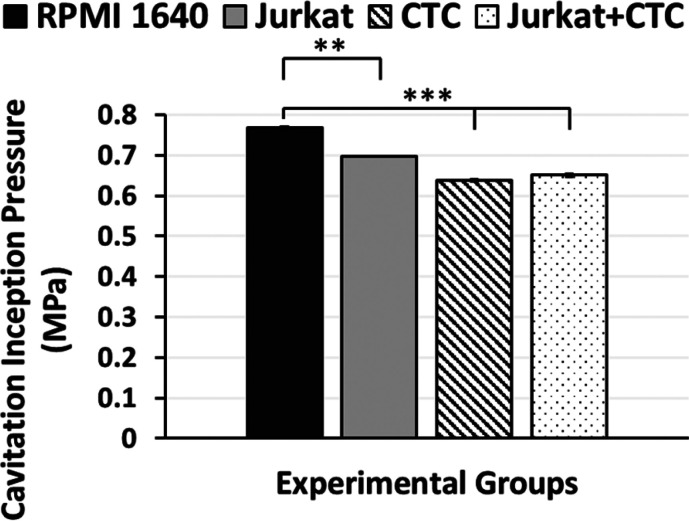
Cavitation inception pressures of different working fluids for the microfluidic device with roughness (Chip-R). The working fluids are RPMI 1640 cell culture medium, Jurkat, CTC, and Jurkat + CTC. Error bars indicate standard deviation.

The microfluidic device (Chip-R) leads to different cavitation inception pressure values compared to the Chip-wo-R for different working fluids (Figure 4). Similar to the previous experiment, the Jurkat cell concentration is 1 × 10^6^ cells/mL for the Jurkat and Jurkat + CTC groups, while the CTC concentration is 300 CTCs/mL for the CTC group and Jurkat + CTC group. Hence, the ratio of CTC to Jurkat is 0.0003. RPMI 1640 has an inception pressure of 0.77 MPa ± 0.02 while the inception pressure for Jurkat becomes 0.69 MPa ± 0.02. The difference between the RPMI group and others is significant. However, there is no significant difference among the Jurkat, CTC, and Jurkat + CTC groups. The CTC group inception pressure is 0.64 MPa ± 0.003, while the inception pressure is 0.65 ± 0.003 MPa for the Jurkat + CTC group.

#### Relationship between Cavitation Inception and Concentration of Cell-CTC Enrichment

**Figure 5:**
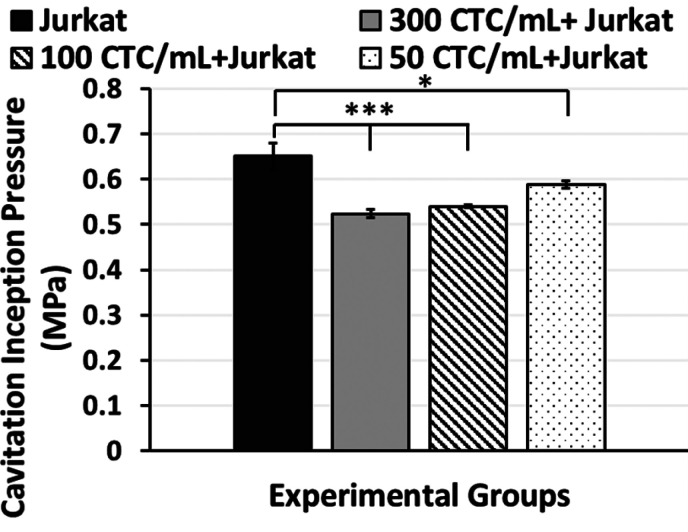
Column graph of the cavitation inception pressure of Jurkat, 300 CTCs/mL + Jurkat, 100 CTCs/mL + Jurkat cells, and 50 CTCs/mL + Jurkat cells.

Based on the results displayed in Figure 5, the inception pressures of all groups containing different amounts of CTCs are lower compared to the group containing only Jurkat cells. The Jurkat cell concentration is 1 × 10^6^ cells/mL for the Jurkat and Jurkat + CTC groups. The CTC concentrations are 300 CTCs/mL, 100 CTCs/mL, and 50 CTCs/mL for the CTC groups. Hence, the ratios of CTC to Jurkat are 0.0003, 0.0001, and 0.00005, respectively. While the average inception pressure of the group containing Jurkat cells is 0.65 MPa ± 0.03, the average inception pressures of the groups containing 300, 100, and 50 CTC per mL are 0.52 MPa ± 0.01, 0.54 MPa ± 0.003, and 0.59 MPa ± 0.009, respectively. No significant pressure difference can be observed for the 300, 100, and 50 CTC groups per mL. If the concentration of CTCs was over 300 per mL, perhaps the decreasing trend in the inception pressure with increasing CTC numbers could have been captured. Moreover, while 300 CTCs/mL and 100 CTCs/mL have a significant difference for the Jurkat groups, the difference between 50 CTCs/mL and the Jurkat group is less significant compared to other groups.

**Figure 6:**
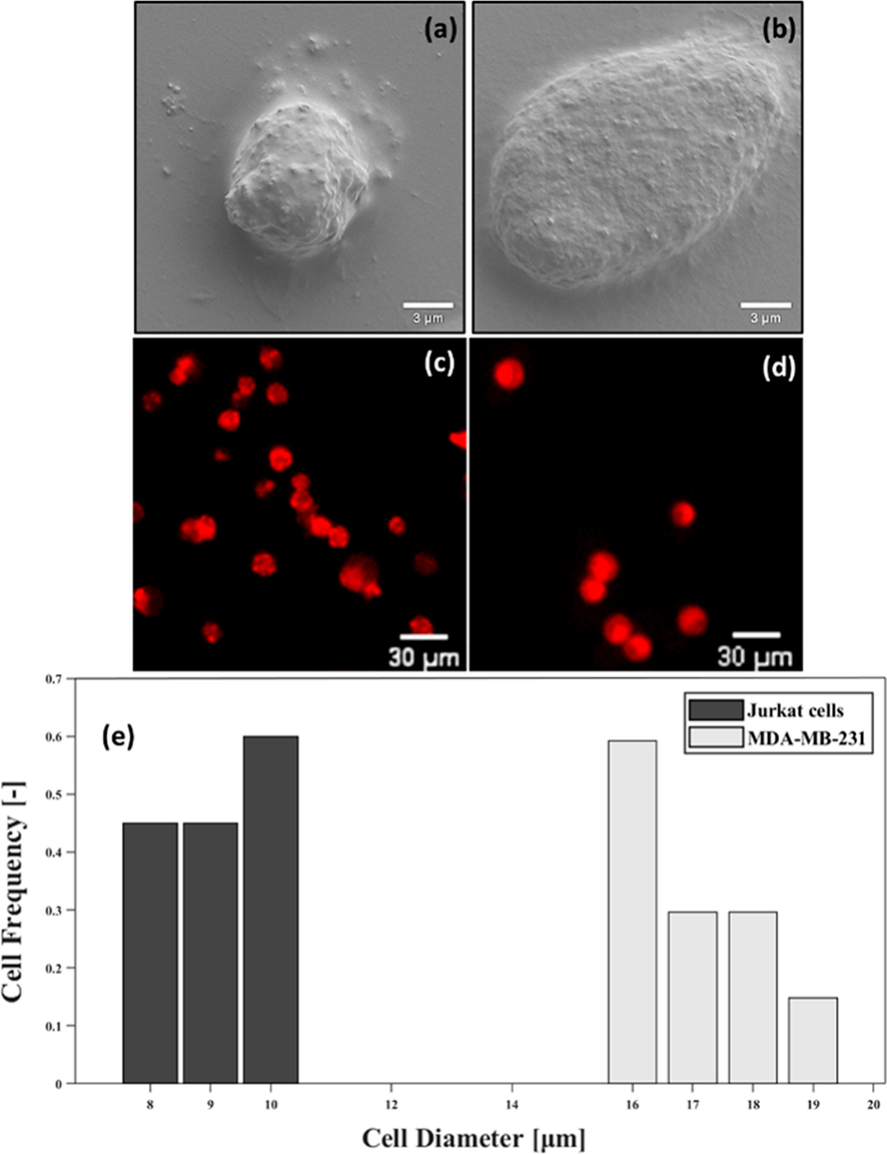
SEM images of the (a) MDA-MB-231 cell line and (b) Jurkat cell line. Fluorescence microscopy images of the cells after staining: Jurkat Cell (c), MDA-MB-231 (d). Size distribution of the Jurkat and MDA-MB-231 cells (e).

The size distribution graph shows that Jurkat cells range from 8 μm to 10 μm, and the obtained sizes of MDA-MB-231 cells range from 16 μm to 19 μm (Figure 6). As a demonstrable proof, the SEM images of Jurkat cells and MDA-MB-231 are shown in Figure 6a–d. A single MDA-MB-231 cell’s diameter was measured as 17.9 μm, while the diameter of the Jurkat cell was 7.1 μm. The average value of these diameter was obtained by taking measurements from different regions of SEM images of both types of cells. MDA-MB-231 cells, which are approximately three times larger in size, affect the cavitation inception behavior and cause an earlier cavitation inception (at considerably lower upstream pressures).

#### Cavitation Inception of Blood Sample

**Figure 7:**
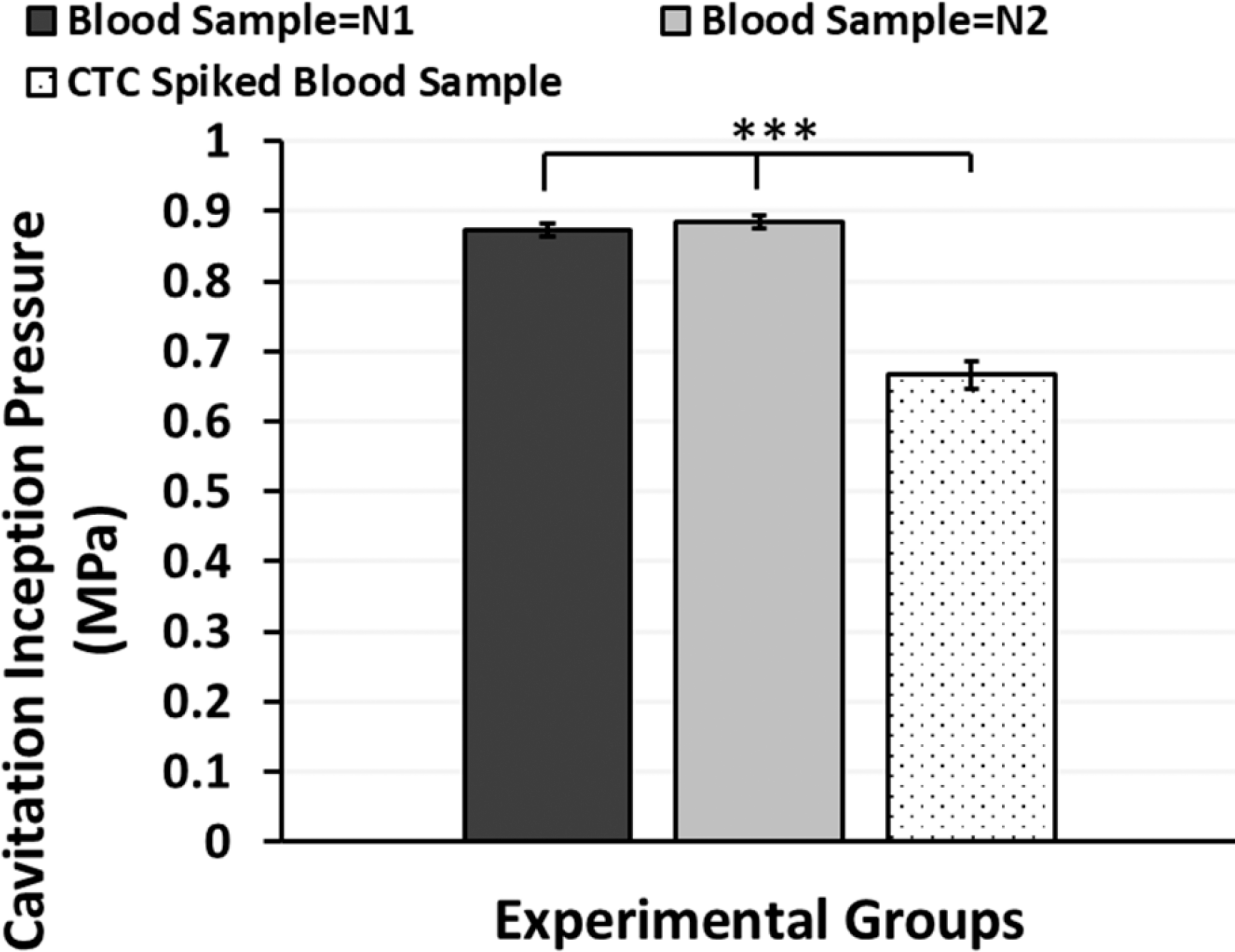
Column graph of the cavitation inception pressures of different blood samples. Blood sample N1 and blood sample N2 represent the different collections of the blood samples from volunteers. The last group is the blood sample with CTC spiked into the N2 group.

The N1 and N2 groups represent healthy blood samples while 300 CTCs/mL is spiked into the other group. The ratio of CTC to WBC is 0.0003. According to the results shown in Figure 7, the inception pressure of the N1 blood sample is 0.87 MPa ± 0.01, and that of the N2 blood sample is 0.88 MPa ± 0.01. Thus, there is no significant difference. However, the CTC-spiked blood sample results in a significantly lower inception pressure, which is 0.67 MPa ± 0.02. The N2 blood sample and the sample with CTC were collected from the same healthy volunteers. Therefore, the concentration and ingredients are considered as the same. Especially, in comparison of the N2 and CTC-spiked groups, the CTC group leads to noticeably earlier cavitation inception. Additionally, the CTC-spiked blood sample also has a significant difference compared to the N1 group.

## Discussion

This study introduces a method which enables rapid detection of the CTCs within minutes. Most of the CTCs have a very short survival time due to several reasons, such as shear stress of flow, anoikis, and extravasation.^26^ In circulation, the limited half-life of CTCs is between 1 and 2.4 h, which implies a short time for detection.^27^ The quick diagnosis capability of this method could detect intravascular cancer cells before their disappearance time, unlike detection methods with specific labels that are more costly and demanding.

This study utilizes cell culture tools and cancer cells-spiked blood during the initial investigation and development phase of CTC detection tools, which is crucial to understand the mechanism of the detection technology in its early stages. On the other hand, the current study aims to detect the presence of CTCs directly from patients’ blood rather than collecting/isolating CTCs for analysis purposes in subsequent analysis. The results show that the Jurkat + CTC group possesses earlier cavitation inception than other groups by creating a more heterogenic environment, which means cancer cells in the sample fluid can be detected by using the cavitation phenomenon on Chip-wo-R (Figure 3). Moreover, group CTC demonstrates more inception pressure reduction than group Jurkat since the CTC group provides more heterogeneous nucleation sites considering the higher surface area it has (Figure 3).

Our results show that the cavitation bubbles’ occurrence is dependent on the shape of the channel and the content of the working fluids. The reduction of inlet pressure required for the inception cavitation in the presence of immersed particles and surface roughness could be explained based on the heterogeneous nucleation theorem. Within a liquid, nucleation stems from the ephemeral microscopic voids created by random molecular motion inside the liquid paving the way for the nuclei to grow and rupture. In heterogeneous nucleation, the voids are originated at the interface of the liquid/solid/vapor intersection of the suspended particles or channel wall. Therefore, the contact angle of the bubbles on the surface of the nucleation site plays an important role in reducing the tensile strength of the liquid. This reduction in tensile strength at the boundaries of the channel wall and suspended particles turns them into potential cavitation bubble nucleation sites, which are called “surface nuclei” and “stream nuclei”, respectively. Thus, the stream nuclei originating from the particles—here the cells—and surface nuclei caused from the sidewall roughness elements are major locations of bubbles’ generation. This also causes the pressure reduction in the inception of the bubbles. Our results for using two sets of microfluidic devices with and without sidewall roughness elements showed that when the dominance of the stream nuclei is preserved with using smooth sidewalls, detection of CTCs from Jurkat cells was enhanced. A reason could be that by suppressing the surface nucleation sites, the presence of CTCs as stream nucleation sites became the sole reason for tensile strength reduction in the liquid.

According to cell size distribution graph (Figure 6e), CTCs have a larger diameter. Thus, CTC-spiked mediums consequently have early cavitation inception (lower upstream pressure) by providing more solid/liquid/vapor interface compared to the Jurkat group without any CTCs. In light of the SEM analysis, the authors identify the difference between the morphological features of the cells, such as size, as the most significant parameter in obtaining a lower cavitation inception value in the group containing CTCs. Our subsequent research studies will focus on determining the effects of the properties of cells, such as stiffness, and working fluids, such as rheology. For this aim, the authors will also evaluate the AFM force measurement analysis and discuss how cell elasticity could affect cavitation inception. The different morphological properties of cells contribute to the change in cavitation inception behavior without utilizing the immunological profile of them. Hence, the HCOC captures CTCs without being affected by heterogeneous cell distribution resulting from the epithelial–mesenchymal transition. However, unlike some current technologies, the HCOC system does not offer isolation, enumeration, and retrieval of CTCs. Instead, it provides only the detection of very rare CTCs in biological fluids such as blood.

The HCOC system can detect the presence of CTC down to 50 CTCs/mL in the experiments carried out so far as an initial study (Figure 5). Even there is a 6-fold difference between the concentrations of 300 CTCs/mL and 50 CTCs/mL; the difference is only 250 CTCs/mL, which is extremely low compared to the total cells (CTC + Jurkat) present in the system. This could be the reason that the difference between the experimental groups containing the lowest and highest CTC concentrations cannot be detected by HCOC. In contrast, the presence of the CTCs can be detected in both groups. In the clinical approach, after the removal of red blood cells from blood, the ratio of CTCs and white blood cells should be considered. In the blood of a healthy person, there are 1–4 million lymphocytes,^28,29^, while, in a patient, the number of CTCs can vary between 1 and 1000 per mL.^4,5^ Therefore, the applicability of the CTC concentration in the clinic is close to 1–1000 CTCs/1–4 million lymphocytes. Accordingly, the authors mimicked a mixture of mononuclear immune cells and CTCs employing Jurkat cells and CTCs, and the ratio is 50–300 CTCs/1 million Jurkat cells. Even though the number of CTCs in the patient’s blood is meager, some studies showed that there are 39 CTCs/2 mL in the nonmetastatic group and 119 CTCs/2 mL in the metastatic group.^30^ The HCOC detection limit is 50 CTCs/mL in this study. This number is close to the number of CTCs in the metastatic group, which implies that the HCOC enables the detection of CTCs in the metastatic group in the range of 50–300 CTCs/mL. In order to increase the detection sensitivity, the configuration of the devices, such as hydraulic diameter, L/d ratio, and surface or sidewall roughness properties, should be further optimized. Achieving a further reduction in the cavitation inception pressure would not destruct cells to a large extent and would offer an opportunity to analyze and characterize rare cells after detection. The cavitation inception results of different CTC concentrations affirm the reliance on a satisfactory level of CTC detection. In the blood, CTCs travel as single cells or in clusters by aggregation, and it was reported that CTC clusters had a higher potential for metastasis.^31,32^ Since CTC clusters consist of aggregates of cells, they are expected to lead to lower cavitation inception and to facilitate detection in HCOC.

Each individual could have different concentrations of protein in blood.^33^ Human blood is composed of cells, lipids, and proteins. Most of this composition is coming from cells, followed by proteins and lipids. The total protein content of a healthy person is 6–8.3 g/dL, whereas the total cholesterol is less than 200 mg/dL (0.2 g/dL). From a clinical biochemist point of view, fluctuations in blood protein levels are more relevant to affect blood viscosity and interfere with biochemical detection processes. Therefore, we performed real sample tests to observe cavitation inception within devices working with blood samples from different volunteers. The results demonstrate that even healthy blood from different individuals brings cavitation inception at approximately the same pressure (N1, N2). The authors believe that the size of the molecules within blood, such as proteins, lipids, is in the nanoscale, which results in no effect in the process of cavitation inception in the proposed detection sensitivity. Moreover, the CTC-spiked blood sample has a very significant difference compared to the same blood sample without CTCs in terms of inception pressure. Here, CTCs demonstrate a dominant effect against blood components comprising the largest component even smaller than CTCs based on their sizes. Hence, the results prove that the HCOC system can detect CTCs in real samples. It is noteworthy to mention that red blood cells’ elimination is generally required for CTC detection procedures. There are many technologies available for their separation including straightforward and conventional methods, such as centrifugation, along with cutting edge technologies.^34^ The elimination of red blood cells results in a mixture of mononuclear immune cells and CTCs.^35^ However, red blood cells could be separated by a straightforward and conventional fashion, such as centrifugation, rather than sophisticated technologies. On the other hand, the amount of blood sample used for each experiment is 200 mL. Integration of a low-cost microphone capable of detecting the cavitation inception noise allows CTC detection even in tiny volumes, which is considered for a more sensitive system. Therefore, the authors believe that more sensitive systems with lower volume could be obtained by a more sensitive detection system.

The current proposed microfluidic CTC detection lab on a chip device with the new concept “HCOC” is therefore a valuable tool by enabling the detection of CTCs, maybe even CTC clusters, in a rapid, cost-effective, and user-friendly fashion.

## Conclusions

A new concept of “hydrodynamic cavitation-on-chip” was proposed here for the detection of CTCs. This method is advantageous since it is label-free and has short on-chip residence times. In this study, cavitating inception of four different fluids—RPMI 1640 medium, the Jurkat group, CTCs, and the Jurkat + CTC group—was employed. The results demonstrated that earlier inception was obtained in the group with CTCs. The significant difference among inception pressures of experimental groups, especially between groups with and without CTCs, led us to benefit from the cavitation phenomenon for CTC detection. The solid/liquid/vapor interface as the main reason for decreasing the tensile strength of a liquid is dependent on the size of floating particles and the contact angle of the interface. The effects of different concentrations of CTCs were assessed to investigate the HCOC concept further. Moreover, blood tests were performed to compare the effect of different blood samples from healthy volunteers on cavitation inception. Blood samples from different individuals have approximately the same cavitation inception, while CTC-spiked blood results in significantly lower cavitation inception. Nonetheless, the microfluidic device design will be further considered, and the effect of cell properties on the system will be investigated in detail in subsequent studies for more advanced and sensitive detection applications of the HCOC system. The number of CTCs (concentration) will be reduced to a lower limit of the counted CTC number in the metastatic cancer group in the literature, and CTC detection utilizing cavitation in this limit will be evaluated. The HCOC concept could achieve early CTC detection so that we expect this inexpensive microfluidic approach to be a facile operation procedure.

## Supporting Information Available

Additional experimental details, materials, and methods, including a schematic image of the fabrication flow and background information about the cavitation phenomenon (PDF)
