# Supplementary material for: Hydrodynamic Cavitation on a Chip: A Tool to Detect Circulating Tumor Cells
Source: ACS Appl Mater Interfaces. 2022 Sep 1;14(36):40688–97. doi: 10.1021/acsami.2c12356 (PMC9478945; doi:10.1021/acsami.2c12356)
Supplement: Supplementary file 1 — am2c12356_si_001.pdf [file am2c12356_si_001.pdf]

Supporting Information

Hydrodynamic Cavitation on a Chip: A Tool to Detect Circulating Tumor Cells

Ilayda Namli, [||,†,‡] Seyedali Seyedmirzaei Sarraf, [||,†,‡] Araz Sheibani Aghdam, [†,‡] Gizem Celebi Torabfam,[†,‡] Ozlem Kutlu, [†,‡,#] Sibel Cetinel,[†,‡,#] Morteza Ghorbani, [‡,#] Ali Koşar, [*,†,‡,#]

† Faculty of Engineering and Natural Sciences, Sabanci University, 34956 Tuzla, Istanbul, Turkey
‡ Sabanci University Nanotechnology Research and Application Center, 34956 Tuzla, Istanbul, Turkey
# Center of Excellence for Functional Surfaces and Interfaces for Nano-Diagnostics (EFSUN), Sabanci University, Orhanli, 34956, Tuzla, Istanbul, Turkey

Corresponding Author: *Email: kosara@sabanciuniv.edu
|| Ilayda Namli, and Seyedali Seyedmirzaei Sarraf contributed equally to this work as the co-first authors

SUPPORTING INFORMATION 1

Experimental Setup Cleaning Protocols

Before the experiments, the container and tubing were washed separately for about ten minutes by passing DI water through them, and the microfluidic device was sonicated for about 15 minutes in acetone before installing the setup. Afterwards, 600 mL of 70% ethanol was passed through the experiment setup at a pressure up to 2.07 MPa. DI water was also passed for three times through the setup at a pressure of 1.38 MPa to remove remaining ethanol. All the above-mentioned protocol was repeated after conducting the tests for each experimental group.

SUPPORTING INFORMATION 2

Microfluidic Device Fabrication Flow

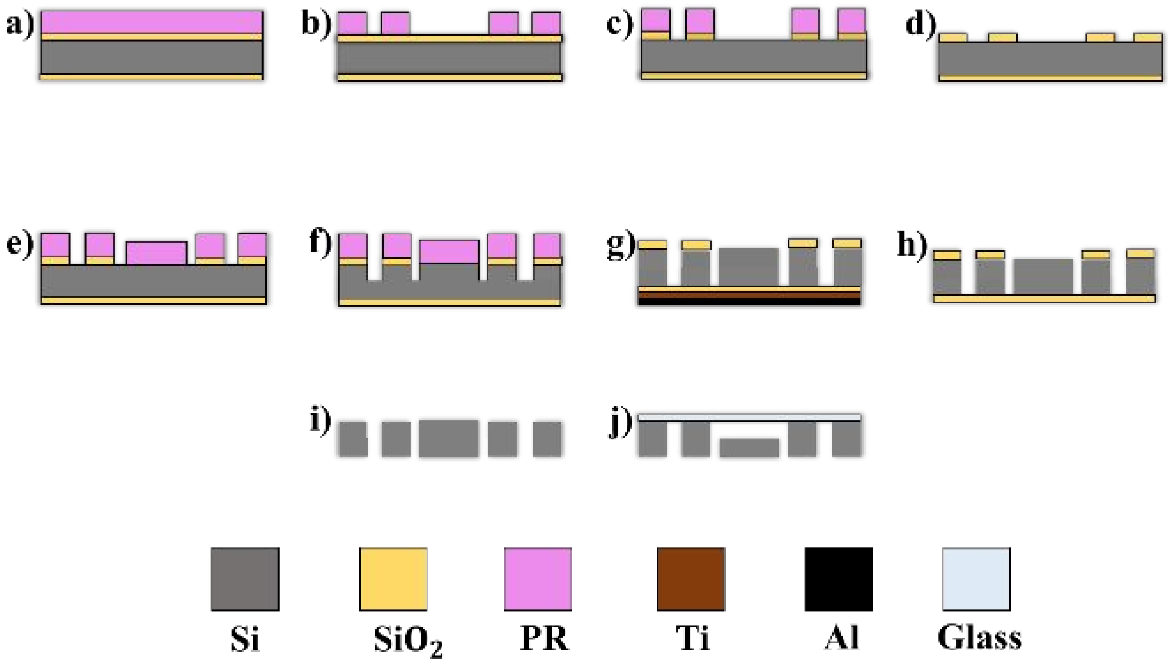

Figure S1. Fabrication flow of microfluidic device without roughness. **a)** Photoresist casting on a silicon dioxide-coated wafer. **b)** Lithography with photoresist coating for channel design. **c)** SiO_2 etching**, d)** photoresist removal, **e)** Lithography for the inlet–outlet and pressure ports. **f)** DRIE for Si to open inlet-outlet and pressure ports. **g**) Resist stripping and Ti, Al coating also DRIE for etching through the wafer **h)** Wet etching of Al and Ti**. i)** Wet etching of SiO_2**. j)** Anodic bonding of the silicon wafer to glass.

SUPPORTING INFORMATION 3

Characterization methods: SEM Sample Preparation

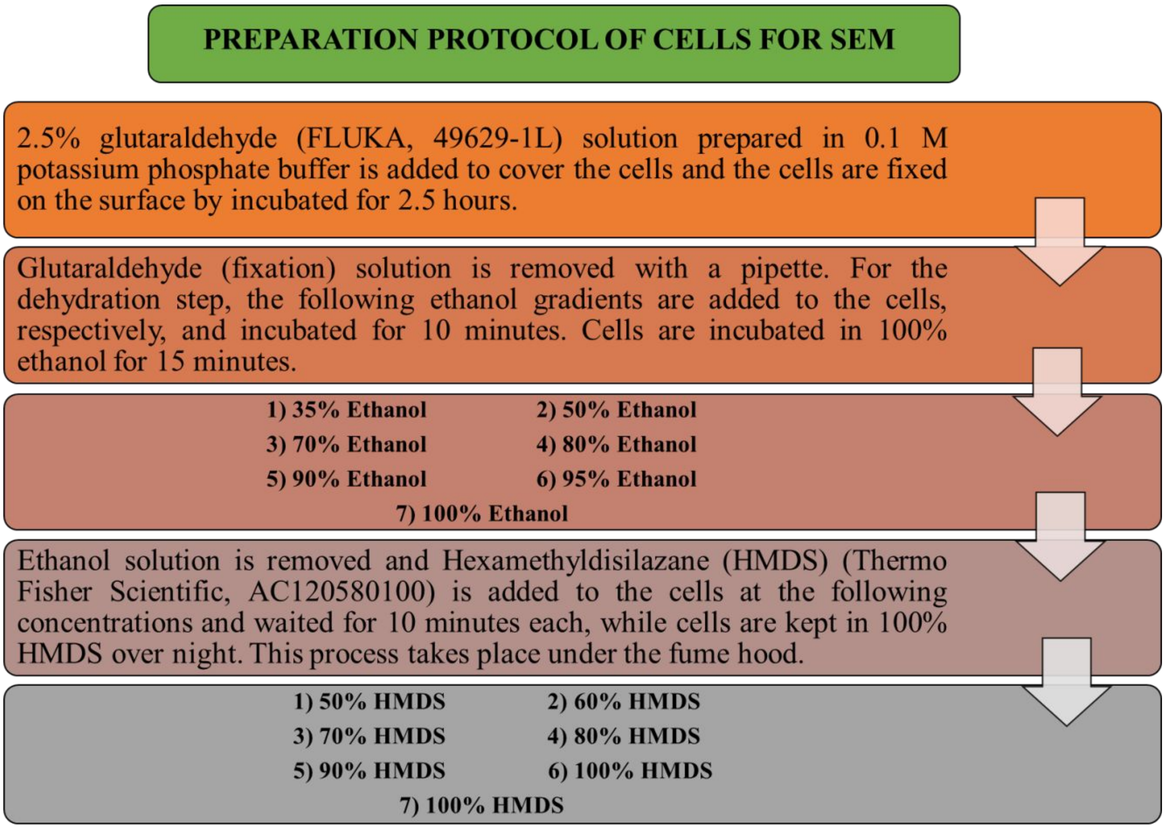

Figure S2. Cell fixation protocol for SEM analysis.

SUPPORTING INFORMATION 4

Flow Cytometry Analysis

Following processing of whole blood samples to isolate WBCs, Hoechst-stained MDA-MB-231 cells were added to WBCs and were characterized using an BD LSRFortessa Cell Analyzer (BD Biosciences). Also, the mixture of Jurkat T cells and Hoechst-stained MDA-MB-231 cells were prepared for the same analysis. MDA-MB-231 cells were prepared in a 10 ug/ml Hoechst (33258) solution in PBS for 15 minutes. 10.000 cells were analyzed using a Pacific Blue channel with 405 nm laser on a BD LSRFortessa flow cytometer. Cells were gated on FSC v SSC. Flow cytometry data were analyzed using the FCS Express 7 Plus (De Novo Software).

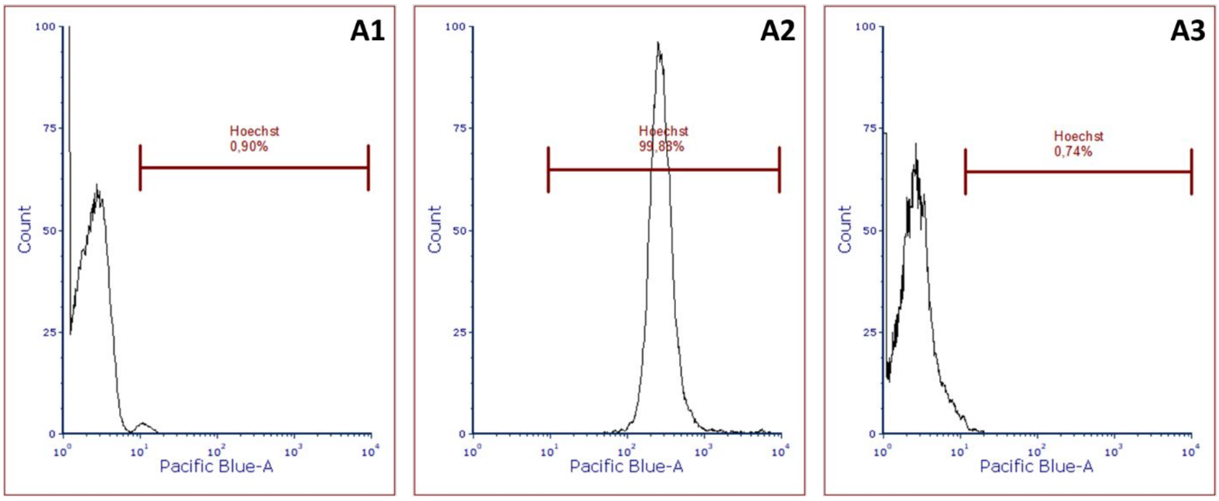

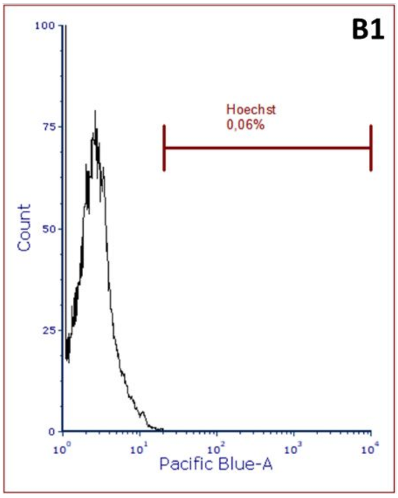

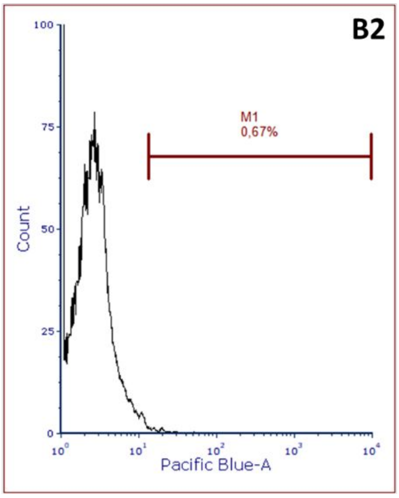

Figure S3. Hoechst staining observation of MDA-MB-231 cells in the mixture with white blood cells or Jurkat T cells. Cells were freshly prepared and processed according to the Hoechst (33258) staining protocol. The ratio of MDA-MB-231 and Jurkat T cells or WBCs was 0.3 %. After gating on live cells, single cells were gated using Pacific Blue channel (405 nm laser) to determine the percentage of Hoechst-stained MDA-MB-231 cells. (A1, A2, A3) Hoechst-stained MDA-MB-231 cells in WBCs from human peripheral blood samples; unstained cells were negative control (A1), only Hoechst-stained MDA-MB-231 cells were positive control (A2), WBCs and MDA-MB-231 mixture (A3). (B1, B2) Hoechst-stained MDA-MB-231 cells in Jurkat T cells; unstained cells were negative control (B1), Jurkat T cells and MDA-MB-231 mixture (B2). Flow cytometry data were analyzed using the FCS Express 7 Plus (De Novo Software).

Flow cytometry analysis is not sufficient to detect MDA-MB-231 cells in the mixture of white blood cells. However, HC detection approach demonstrates the ability to detect CTCs up to 50 per mL.
